# A process‐oriented perspective on pre‐service teachers' self‐efficacy and their motivational messages: Using large language models to classify teachers' speech

**DOI:** 10.1111/bjep.12779

**Published:** 2025-04-28

**Authors:** Olivia Metzner, Yindong Wang, Wendy Symes, Yizhen Huang, Lena Keller, Gerard de Melo, Rebecca Lazarides

**Affiliations:** ^1^ Department of Educational Sciences University of Potsdam Potsdam Germany; ^2^ Chair of Artificial Intelligence and Intelligent Systems Hasso‐Plattner‐Institute/University of Potsdam Potsdam Germany; ^3^ Department of General, Intercultural and International Comparative Education as well as Educational Psychology (EW1) University of Hamburg Hamburg Germany; ^4^ Institute for Psychology in Education, University of Münster Münster Germany; ^5^ Department of Research on Teaching and Teacher Education Kiel University Kiel Germany; ^6^ Institute for Psychology of Learning and Instruction (IPL), Kiel University Kiel Germany; ^7^ Science of Intelligence, Research Cluster of Excellence Berlin Germany

**Keywords:** artificial intelligence, large language models, motivational messages, self‐determination theory, teacher self‐efficacy

## Abstract

**Background:**

Recent studies have examined the relation between teacher motivation, motivational messages and student learning but are limited to an achievement‐related context, primarily using survey data. Moreover, our understanding of the relation between various teacher characteristics, such as teacher self‐efficacy (TSE), and their motivational message use remains limited.

**Aims:**

Our study tested whether teacher speech can be classified into self‐determination (SDT)‐based motivational messages and reliably assessed with a large language model (LLM). Additionally, we analysed the relation between pre‐service TSE and their motivational message use.

**Sample:**

For our first aim, we used human‐rater annotations from 119 pre‐service teachers' classroom recordings. For our second aim, we used data from 103 pre‐service teachers (52.69% female; *M*_age_ = 22.98, *SD*_age_ = 3.26, *Min*_age_ = 19, *Max*_age_ = 34) who participated in a survey and were video‐recorded while teaching.

**Methods:**

First, we manually classified pre‐service teachers' motivational messages based on transcripts and used human‐rater annotations to fine‐tune an LLM. Second, we analysed the relation between pre‐service TSE and motivational message use.

**Results and Conclusions:**

The fine‐tuned LLM demonstrated promising performance in assessing SDT‐based motivational messages but needs further refining to assess thwarting messages. The analysis with human annotation showed that pre‐service TSE for classroom management positively affected the frequency of relatedness‐supportive messages. Pre‐service TSE for student engagement increased the likelihood of never using a competence‐ or relatedness‐thwarting message. Pre‐service TSE for instructional strategies reduced the frequency of autonomy‐supportive messages. LLM‐based analyses showed slightly different results but did not contradict human annotation‐based analyses.

## INTRODUCTION

Teacher motivational messages are central for students' achievement and motivation (Putwain & Remedios, 2014; Reeve & Jang, 2006; Santana‐Monagas et al., 2022); however, current research has certain theoretical and methodological limitations. Our study aimed to expand on existing research with regard to two objectives.

First, until now, research has primarily focused on teacher motivational messages in achievement‐related contexts (e.g., Alqassab & León, 2024; Putwain et al., 2017). Our study aimed to complement existing research by focusing on teachers' motivational messages that support or thwart students' feelings of autonomy, competence and relatedness – the three basic psychological needs essential for enhancing intrinsic motivation according to self‐determination theory (SDT; Deci & Ryan, 2002; Ryan & Deci, 2020). Referring to prior work on teachers' SDT‐based messages (Reeve & Jang, 2006), we argue that identifying teachers' messages that support or thwart these needs enhances our understanding of motivational processes in class. Additionally, research on teacher motivational messages has predominantly relied on survey data (Putwain & Symes, 2011; Santana‐Monagas & Núñez, 2022), although there are a few recent exceptions (e.g., Falcon et al., 2023; Reeve & Jang, 2006). We addressed this gap by recording pre‐service teachers in authentic classroom settings and tested whether an AI‐based method (large language model; LLM) can reliably categorise teachers' motivational messages. To achieve this, we developed and tested a new coding scheme to assess teachers' SDT‐based motivational messages and human raters used this coding scheme to annotate teacher classroom transcripts. The annotated data were then used to fine‐tune a pre‐trained LLM and evaluate whether the LLM could reliably assess teachers' motivational messages.

Second, research that outlines the processes taking place between teachers' motivation and their teaching behaviours are under‐researched. Studies on teacher self‐efficacy (TSE) have shown that TSE is a critical factor for effective teaching (for an overview, see Klassen & Tze, 2014; Lauermann & ten Hagen, 2021; Lazarides & Warner, 2020); however, the specific actions teachers with high self‐efficacy take to achieve quality teaching remain unclear. Theoretical frameworks have posited that teacher motivation, including TSE, is linked to teaching behaviours (Lazarides et al., 2024; Tschannen‐Moran et al., 1998) through, for example, the use of verbal messages. Yet, there is limited research investigating how specific motivational beliefs, like TSE, are related to specific teacher behaviours, such as motivational messages. In our study, we used the model of Tschannen‐Moran et al.'s (1998) on TSE and its linkages to teaching performance and combined it with work on SDT‐based teacher messages (Reeve & Jang, 2006) to investigate a specific type of teacher action – namely, the use of motivational messages – and examined how TSE predicts teacher motivational message use in the classroom. We used human‐rated teacher transcript data to explore the longitudinal links between pre‐service teacher‐reported TSE at the beginning of the semester and pre‐service teachers' motivational message use mid‐semester. Furthermore, we conducted an additional analysis in which we used the fine‐tuned LLM predictions of supportive messages to investigate their relation with pre‐service TSE beliefs.

## MOTIVATIONAL MESSAGES IN THE CLASSROOM

Teaching behaviours comprise both the verbal and nonverbal behaviours of teachers in class (Liu, 2021). In this study, we focused on teacher motivational messages as a type of teachers' verbal behaviour. Teacher motivational messages can be defined as advisory messages from teachers that leverage specific types of motivation to encourage students' engagement in school‐related activities (Santana‐Monagas et al., 2022). Various studies have examined the relation between teacher motivational messages and student outcomes, such as the use of teachers' gain‐ and loss‐framed motivational messages (e.g., Falcon et al., 2023; Putwain et al., 2017). Gain‐framed messages refer to teacher messages that highlight students' potential gain or attainment from following a certain course of action, while loss‐framed messages emphasise what can be lost or not attained (Putwain et al., 2021). Existing empirical work has shown that teachers' gain‐framed messages with an autonomous motivational appeal indirectly predicted students' academic performance through students' autonomous motivation cross‐sectionally (Santana‐Monagas et al., 2022). Putwain et al. (2017) showed that students who appraised fear appeals–loss‐framed motivational messages that emphasise the negative consequences of failure–as threating, exhibited lower examination performance over time due to reduced behavioural engagement. In addition to gain‐ and loss‐framed messages, some studies have investigated other types of supportive teacher motivational messages. In a quasi‐experimental study, von der Embse et al. (2015) demonstrated that university students achieved higher exam scores when efficacy appeals–messages emphasising an individual's ability to achieve an outcome – were delivered prior to an exam. In contrast, university students who received fear appeals before an exam performed worse (von der Embse et al., 2015). Alqassab and León (2024) showed that student‐reported teacher reassuring messages, which demonstrate emotional support, hope and optimism, were positively and cross‐sectionally related to students' intrinsic motivation. However, although researchers have investigated different types of motivational messages, they have mainly focused on motivational messages used in achievement‐related contexts. This means the messages either highlighted the consequences of completing or not completing a task (e.g., Putwain et al., 2017; Santana‐Monagas et al., 2022) or were specifically used and tested in the context of exam preparation (e.g., Alqassab & León, 2024; von der Embse et al., 2015).

Although extensive research exists on gain‐ versus loss‐framed messages, relatively few studies have focused on messages that promote students' intrinsic motivation. On a theoretical level, SDT (Deci & Ryan, 2000, 2002) proposes that teaching behaviours that address students' innate needs for autonomy, competence and relatedness are central to fostering students' interest and intrinsic forms of motivation to learn. Autonomy is defined as a person's sense of initiative and personal ownership over a task, competence can be understood as a feeling of mastery and confidence in one's ability to succeed and develop, and relatedness is conceptualised as a sense of belonging (Ryan & Deci, 2020). In an experimental study, Reeve and Jang (2006) randomly assigned pre‐service teachers to either a teacher or student role and investigated the relation between autonomy‐supportive and autonomy‐controlling instructional behaviours and students' perceived autonomy in a one‐on‐one, 10‐min classroom setting. The results showed that offering encouragement–classified in this study as an autonomy‐supportive instructional behaviour–was positively and significantly associated with students' perceived autonomy. In contrast, autonomy‐controlling instructional behaviours, such as asking controlling questions and using “should” or “ought to” statements, were negatively related to students' perceived autonomy (Reeve & Jang, 2006). Recently, Ahmadi et al. (2023) successfully applied SDT to classify teachers' verbal and nonverbal supportive and thwarting teaching behaviours in class. The classification system assigns SDT‐based behaviours to six categories: autonomy‐, competence‐ and relatedness‐supportive or thwarting behaviours. The study included verbal and nonverbal teaching behaviours. In this study, we utilised Ahmadi et al.'s (2023) classification system and extracted teachers' verbal cues to develop an SDT‐based motivational message scheme (for more details, see the Measures section).

## **M**ETHODOLOGICAL CONSIDERATIONS OF TEACHER MOTIVATIONAL MESSAGE RESEARCH

Until now, research on teacher motivational message use in class has predominately relied on students' reports of teachers' messages (Putwain & Symes, 2011; Santana‐Monagas & Núñez, 2022). Although students' reports can provide insights into classroom activities, they may be shaped by extraneous factors, such as individual interest in the subject or social desirability effects, which can limit their objectivity and may, therefore, fail to present a comprehensive view of classroom dynamics (King & Bruner, 2000). As an alternative, researchers could use transcripts of video‐recorded authentic lessons to assess teachers' motivational messages. However, such an approach is highly resource‐intensive, requiring the training of human raters to manually annotate large quantities of data (Cash et al., 2012). One possible solution to this problem might lie in new methods, such as LLM, that enable researchers to annotate text automatically based on previously defined criteria. LLMs are artificial intelligences (AI) with a large number of parameters trained on extensive corpora to interpret, generate, and process human language (Myers et al., 2024). LLMs can be adjusted to specific demands through fine‐tuning, adapting a pre‐trained model to specific tasks using a smaller, specialised data set, allowing quick adjustment to new tasks and data distributions. Unlike traditional machine learning (e.g., simple text mining), LLMs capture contextual word meanings rather than treating words as having fixed meanings (Demszky et al., 2023). LLM‐based research on processing natural language has gained multidisciplinary interest (Ding et al., 2024), including educational research (Yan et al., 2024). Falcon and León (2024), for example, fine‐tuned the Ada version of GPT‐3 and developed a two‐stage model to identify and classify teachers' engaging messages into gain‐ and loss‐framed categories. Tan and Demszky (2023) showed that an LLM that was fine‐tuned with teacher classroom transcript data validly assessed teacher classroom‐management language and punitiveness. Following this approach, in our study, we used a newly developed coding scheme based on Ahmadi et al. (2023) to manually identify teachers' SDT‐based motivational messages from classroom videos. These messages were classified by human raters into categories of autonomy‐supportive, competence‐supportive, relatedness‐supportive, autonomy‐thwarting, competence‐thwarting and relatedness‐thwarting messages. Just like Falcon and León's (2024) and Tan and Demszky's (2023) studies, we then used the human‐rated data to fine‐tune an LLM with the aim of reliably assessing teachers' motivational messages.

## TEACHER SELF‐EFFICACY AND MOTIVATING TEACHING BEHAVIOURS

Although there has been much research examining the consequences of motivational messages for student outcomes (e.g., Putwain & Symes, 2011; Santana‐Monagas et al., 2022), less is known about the factors that influence the type and quantity of motivational messages used by teachers. We addressed this gap in our study by examining how teachers' motivational beliefs, specifically their self‐efficacy for teaching, relate to their motivational message use in class. TSE refers to teachers' perceptions of their ability to achieve desirable student learning and engagement outcomes, even with students who struggle or feel unmotivated (Tschannen‐Moran & Hoy, 2001). TSE can be categorised into three dimensions: TSE for instructional strategies, for classroom management and for student engagement. *Teacher self‐efficacy for instructional strategies* defines teachers' beliefs in their ability to implement various instructional methods that target student learning, such as providing alternative explanations or adjusting the level of challenge (Pfitzner‐Eden et al., 2014; Zee et al., 2017), and is positively associated with teacher‐reported cognitive activation, classroom management and emotional support (Lazarides & Schiefele, 2021). *Teacher self‐efficacy for classroom management* describes teachers' beliefs in their ability to maintain order in the classroom, for instance, through the introduction of classroom rules or effective management of disruptive student behaviour (Emmer & Stough, 2001; Pfitzner‐Eden et al., 2014) and has been shown to be longitudinally and positively related to student‐rated class‐level monitoring (Hettinger et al., 2021). *Teacher self‐efficacy for student engagement* refers to teachers' beliefs in their ability to motivate students, particularly those who feel unmotivated (Pfitzner‐Eden et al., 2014; Tschannen‐Moran & Hoy, 2001), and is positively associated with teacher‐reported classroom climate (Fackler et al., 2021) and longitudinally and positively related to class‐level student‐rated emotional support (Lazarides et al., 2023). Summarising these empirical results, it can be assumed that TSE for classroom management fosters teaching behaviours that promote structure and discipline in class, whereas TSE for student engagement promotes teaching behaviours that foster a positive social climate in class. TSE for instruction is not well researched (see Kuusinen, 2016), and thus no clear pathway to specific teaching practices has been empirically identified.

Until now, research examining the link between TSE and need‐supportive behaviour is still quite rare. Holzberger and Prestele (2021) showed on a school‐ and teacher‐level that TSE for instruction was positively and cross‐sectionally related to teachers' use of cognitive activating behaviour. Cognitive activation can be expressed, for instance, through the provision of challenging tasks and questions (Praetorius et al., 2018), which can be considered as a form of competence‐supportive behaviour (Ahmadi et al., 2023). TSE for classroom management was positively and cross‐sectionally related to observer‐rated emotional support (Ryan et al., 2015). Examples of emotional support include showing interest in students' points of view (Praetorius et al., 2018), which is a component of relatedness‐supportive behaviour (Ahmadi et al., 2023). Regarding TSE for student engagement, as one of the few studies, Putwain and von der Embse (2018) found that teachers with lower self‐efficacy for student engagement more frequently used fear appeals. Fear appeals can be understood as a type of need‐thwarting teaching behaviour using derogatory language (Ahmadi et al., 2023).

Referring to such prior work, we propose that the different dimensions of TSE in class are related to teachers' motivational messages – which then over time may manifest in certain need‐supportive or need‐thwarting teaching behaviours. More concretely, we assume that TSE for instructional strategies is linked to teachers' motivational messages that promote cognitive challenges (e.g., competence support), but would be unrelated or negatively related to messages that focus on motivation enhancement only (autonomy or relatedness support). TSE for classroom management could be assumed to foster the establishment of clear communication and rules in the learning environment by strengthening interpersonal relationships (e.g., relatedness support). TSE for student engagement could be assumed to foster motivation‐enhancing messages (e.g., autonomy or relatedness support) and reduce the use of messages that undermine motivation (e.g., relatedness thwarting).

**FIGURE 1:**
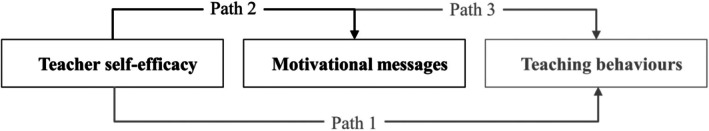
Hypothesised model to examine the process linking teacher self‐efficacy and teaching behaviours.

Theoretical work on teachers' self‐efficacy posits that TSE affects their goal setting, effort and persistence, ultimately shaping their teaching behaviour in class (Tschannen‐Moran et al., 1998). Following this approach, we hypothesised that internal motivational teaching characteristics, more concretely, TSE, would affect teachers' externally displayed motivational messages – which we interpret as an expression of their efforts to teach (Flintcroft et al., 2017; Putwain & Roberts, 2012; Putwain & Symes, 2014). Based on this theoretical background, we introduce a hypothesised model that is illustrated in Figure 1. In this study, we only examine the first part of the model, depicted in solid black lines in Figure 1.

## THE PRESENT STUDY

Against the background of theoretical work on SDT and TSE, and based on prior research examining teachers' motivational messages, we examined the following research questions and hypotheses:

Can pre‐service teachers' SDT‐based motivational messages be reliably assessed with a classification system and with AI‐based methods, namely LLMs?

We hypothesised that teachers' motivational messages could be classified into six distinct categories based on SDT (Ahmadi et al., 2023; Deci & Ryan, 2002) and that a fine‐tuned LLM would reliably assess these categories.

How does pre‐service TSE for instructional strategies, classroom management and student engagement at the beginning of the semester (T_1_) relate to their use of SDT‐based motivational messages mid‐semester (T_2_)?

We hypothesised that pre‐service TSE for instructional strategies at the beginning of the semester (T_1_) would have a positive effect on competence‐supportive messages used in class mid‐semester (T_2_), but would be unrelated or negatively related to autonomy‐ or relatedness‐ supportive messages used in class mid‐semester (T_2_).

We hypothesised that pre‐service TSE for classroom management at the beginning of the semester (T_1_) would have a positive effect on relatedness‐supportive messages used in class mid‐semester (T_2_).

We hypothesised that pre‐service TSE for student engagement at the beginning of the semester (T_1_) would have a positive effect on autonomy‐ and relatedness‐supportive messages used in class mid‐semester (T_2_), as well as a negative effect on relatedness‐thwarting messages used in class mid‐semester (T_2_).

In addition to the second research question, we conducted further analyses to explore the potential of the fine‐tuned LLM as a classification tool for assessing pre‐service teachers' supportive messages and relating them to their self‐efficacy beliefs. However, these analyses cannot be generalised, as we used the same transcript data to fine‐tune the LLM, which may have introduced bias. We therefore analysed the following additional research question: How does pre‐service TSE for instructional strategies, classroom management and student engagement at the beginning of the semester (T_1_) relate to the LLM's prediction of pre‐service teachers' SDT‐based motivational messages mid‐semester (T_2_)?

## METHODS

### Sample

**FIGURE A1:**
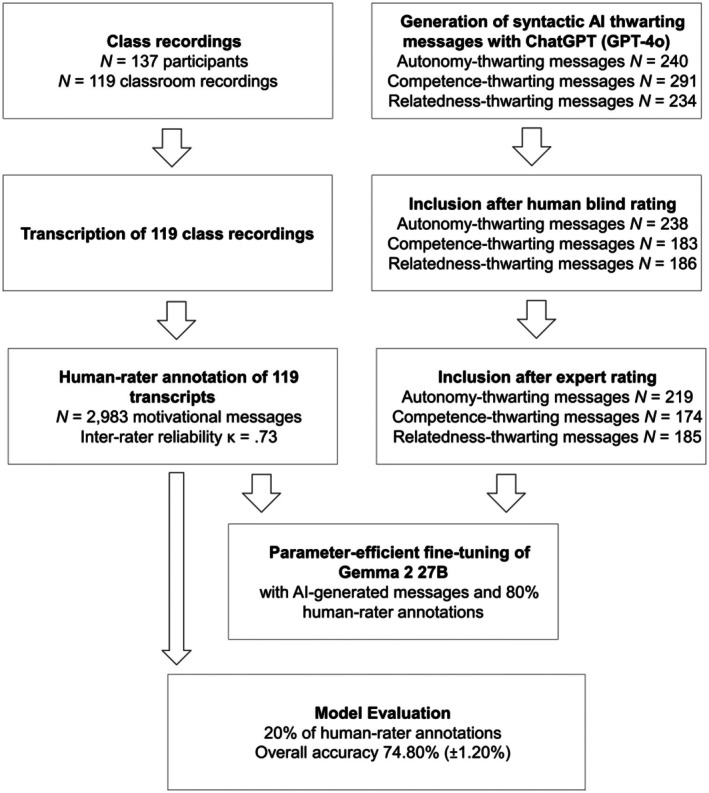
Pipeline Research Question 1.

**FIGURE A2:**
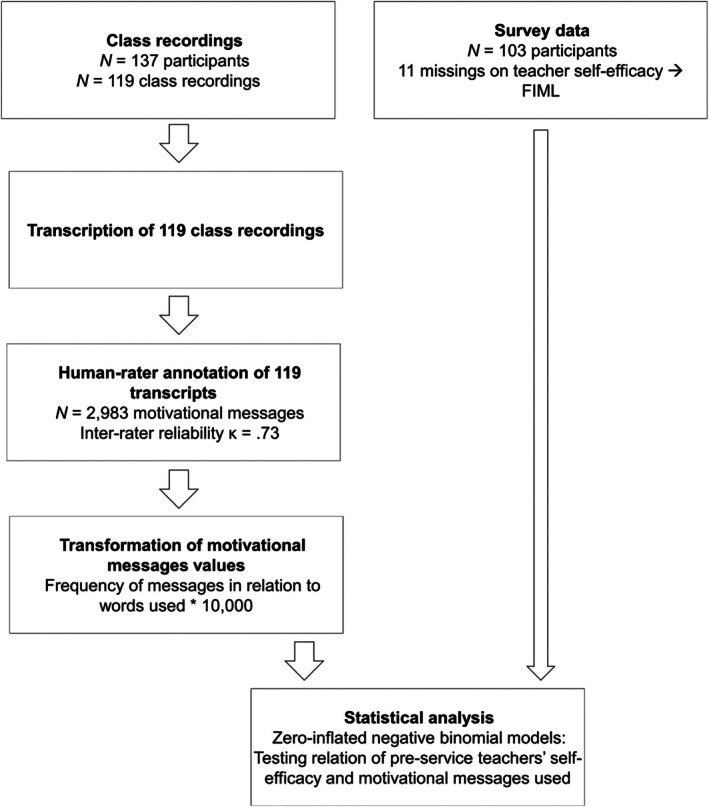
Pipeline Research Question 2.

In this study, we investigated the above two research questions with two cohorts of pre‐service teachers. Figures A1 and A2 in Appendix A illustrate the pipeline of both research questions. To evaluate the reliability of an LLM for assessing pre‐service teachers' motivational messages, video data from 119 recorded lessons of pre‐service teachers were used. To investigate the relations between pre‐service TSE beliefs and their use of motivational messages, a sample of 103 pre‐service teachers (52.69% female; *M*_age_ = 22.98, *SD*_age_ = 3.26, *Min*_age_ = 19, *Max*_age_ = 34) was considered. These pre‐service teachers had video‐recorded their lessons and completed a survey on their motivational beliefs, except for 10 participants who did not fill out the questionnaire. Pre‐service teachers were on average in their fifth semester (*SD* = 1.81, *Min* = 2, *Max* = 15) and the majority were born in Germany (90.55%). Of this sample, the three most studied subjects were German (15.20%), English (12.25%) and Physical Education (9.31%). The study obtained ethical approval from the university ethics committee (protocol no. 41/2023).

### Study design and procedure

The study was conducted in a Bachelor's‐level course for pre‐service teachers in the field of Educational Science at the end of their Bachelor program. The course focused on motivation‐enhancing teaching and was offered 3–4 times per semester for 90 min per week. In the first weeks of the semester, participants learned about teaching quality and motivational strategies and observed a lesson in an assigned secondary school. Afterwards, pre‐service teachers were matched with a teacher in a cooperating school, developed one lesson they would teach in that school, and presented the lesson in the Bachelor course. In the middle of the semester, all participants taught their lessons in the school and video‐recorded the lesson, depending on the data protection regulations at the participating schools. For this study, we only included video and survey data from pre‐service teachers who consented to their data being used for research purposes. The duration of the teaching sessions and classroom recordings ranged from 45 to 90 min, depending on the specific school where the pre‐service teachers conducted their lessons (see Hußner et al., 2024). In total, 119 lessons were video‐recorded. The three most frequently taught subjects were English (21.90%), Mathematics (12.41%) and German (10.95%).

### Measures

The study featured a longitudinal design with two measurement occasions. Pre‐service TSE was assessed at the beginning of the semester (T_1_). Pre‐service teachers' motivational messages were annotated using transcripts of their lessons (T_2_).

#### Teacher self‐efficacy

TSE at the beginning of the semester (T_1_) was assessed using an adapted form of the German version (Pfitzner‐Eden et al., 2014) of the Teacher Efficacy Scale (Tschannen‐Moran & Hoy, 2001). The scale divides TSE beliefs into self‐efficacy for instructional strategies (e.g., “I am confident I can find an alternative explanation or example if learners do not understand something”), for classroom management (e.g., “I am confident in my ability to control disruptive behaviour in the classroom”) and for student engagement (e.g., “I am convinced that I can motivate the learners who have little interest in the lessons”). Response options ranged from 1 (*strongly disagree*) to 6 (*strongly agree*). Reliabilities of TSE dimensions and inter‐rater reliabilities of motivational messages were acceptable. Descriptive statistics for the scale are reported in Table 1.

**TABLE 1:** Descriptive statistics for pre‐service teacher self‐efficacy at the beginning of the semester.

Teacher self‐efficacy	*N*_items_	*M* (*SD*)	Range	*N*_*participants*_	Cronbach's α
TSEIS	4	4.71 (0.60)	3–6	92	.73
TSECM	4	4.42 (0.70)	2–6	92	.84
TSESE	4	4.74 (0.56)	4–6	91	.72

Abbreviations: TSEIS, Teacher self‐efficacy for instructional strategies; TSECM, Teacher self‐efficacy for classroom management; TSESE, Teacher self‐efficacy for student engagement.

#### STD‐based motivational messages

We developed a coding scheme based on the Teachers' Motivational Behaviour classification system by Ahmadi et al. (2023), who defined 57 sub‐categories that describe verbal and nonverbal SDT‐based teaching behaviours in detail. We adapted and summarised Ahmadi et al.'s (2023) sub‐categories for verbal behaviours. We thereby reduced the scheme to the following six main categories: autonomy‐supportive messages, competence‐supportive messages, relatedness‐supportive messages, autonomy‐thwarting messages, competence‐thwarting messages and relatedness‐thwarting messages. Our coding scheme differentiated these six main categories into 23 sub‐categories that were mainly used to provide detailed definitions of the six main categories. For human annotations, we only used the six main categories. The detailed coding scheme is reported in the supplemental material. The transcription of the video recordings was carried out using the AI‐based software DaVinci Resolve Studio (Blackmagic Design, 2023) and Panopto (Panopto, 2024) and was subsequently proofread by research assistants. The transcripts were annotated on a sentence‐by‐sentence basis. One research assistant and one PhD student annotated the transcripts. The raters received a training and regularly met to discuss ratings. Regular meetings and adjustment of the rating process took place until 30 transcripts (25.21%) were doubly annotated with an inter‐rater reliability of κ = .73. The remaining 89 transcripts were annotated by only one rater. The inter‐rater reliability for the main categories is reported later in the Results section.

### Adapting an LLM to assess teachers' motivational messages

To address our first research question, we fine‐tuned an LLM using human‐annotated examples. Initially, we conducted several experiments with various open‐source LLMs. Further details on the initial experiments can be found in Appendix B. The Gemma 2 27B model (Gemma Team, 2024) from Google yielded the best performance in these preliminary tests, and we proceeded with further fine‐tuning of Gemma 2 27B. The Gemma 2 27B model is available for download and can be deployed on local infrastructure. The fine‐tuning and evaluation took place in three steps. Figure A1 in Appendix A displays the pipeline of the fine‐tuning and evaluation of the model.

First, we created a training set to fine‐tune the model and a test set to evaluate its performance. The training and test sets included examples from human‐rated motivational messages as well as out‐of‐domain data, which consist of messages that cannot be assigned to any motivational message category. For the training set, we randomly considered 80% of the human‐annotated data, while the remaining 20% were held back and assigned to the test set. However, due to natural imbalances in the distribution of teacher messages within the transcripts, certain message categories – particularly thwarting messages – were underrepresented (see Results chapter). Fine‐tuning an LLM with imbalanced data could result in lower model performance. To address this issue, we additionally implemented a data augmentation strategy by generating additional thwarting messages using ChatGPT (GPT‐4o). Specifically, we designed instructional prompts tailored to the sub‐categories of thwarting messages, and ChatGPT generated new examples based on these prompts. Before integrating the syntactic messages into the training set, we calculated the inter‐rater agreement between AI ratings and human ratings, yielding agreement levels of 99.17% for autonomy‐thwarting messages, 62.89% for competence‐thwarting messages and 79.49% for relatedness‐thwarting messages. Messages with no agreement were excluded. Moreover, two former teachers evaluated the remaining messages for authenticity, and any messages deemed “too artificial” were excluded. The final set of validated messages was then incorporated into the training set (for a more detailed explanation, see Metzner et al., 2025).

Second, we fine‐tuned and evaluated the model using five independent train‐test splits. To optimise computational efficiency, we employed parameter‐efficient fine‐tuning (PEFT) with the LoRA adapter (Hu et al., 2021). To address class imbalance, we additionally applied class‐balanced weighting, adjusting loss contributions based on sample frequency (Cui et al., 2019). The data distribution, including the number of examples per category from human annotations, AI‐generated messages and the training and testing sets, is presented in Table 2.

**TABLE 2:** Data distribution of human‐annotated, AI‐generated messages and samples per training and test sets.

Motivational messages	*N*_human‐rater annotated messages_	*N*_AI‐generated messages_	*N*_training data set_ ^a^	*N*_test data set_ ^b^
Autonomy support	946	–	753.00	193.00 (±13.80)
Competence support	879	–	707.20	171.80 (±7.30)
Relatedness support	641	–	511.80	129.20 (±11.70)
Autonomy thwarting	286	219	445.40	59.60 (±3.20)
Competence thwarting	57	174	218.20	12.80 (±1.70)
Relatedness thwarting	174	185	328.40	30.60 (±2.60)
Out‐of‐domain data	1,500^c^	–	1200.00	300.00 (±6.00)

^a^ The number of training samples is an estimate. The exact number was not tracked intentionally during the experiments.

^b^ The number of test samples is the average number of samples per category across all five testing splits.

^c^ Out‐of‐domain data (data that cannot be classified into the motivational messages scheme) were only annotated for randomly selected segments from 15 transcripts to obtain a sufficient number of training and testing examples. However, the approximate ratio of out‐of‐domain data among transcript data is 95%.

Third, we assessed the model's predictions by comparing them to the held‐back test set. The model was evaluated across all five runs, and the average performance was calculated for each message category. To evaluate the fine‐tuned model, a comprehensive statistical analysis was conducted, which assessed its accuracy and reliability in classifying motivational messages. Key evaluation metrics included precision, recall, F1‐score, specificity and accuracy, calculated using Python library scikit‐learn (Pedregosa et al., 2011). Precision measured the proportion of correctly identified positive instances among all instances identified as positive. Recall assessed the proportion of correctly identified positive instances among all actual positive instances, whereas specificity measured the proportion of correctly identified negative instances relative to all actual negative instances. The F1‐score, the harmonic mean of precision and recall, offered a balanced measure of the model's performance (Chinchor, 1992). Accuracy represented the overall proportion of correct predictions, including both positive and negative instances (Hossin & Sulaiman, 2015). The F1‐score's interpretation depends on the model's specific task and domain. In this study, we drew on previous research that fine‐tuned an LLM with teacher messages and reported F1‐scores between 0.60 and 0.89 (Falcon & León, 2024; Tan & Demszky, 2023). Guided by these findings, we set a minimum F1‐score threshold of 0.70 to ensure the reliability of our model's performance. Statistical analyses assessing the model's performance, facilitated by scikit‐learn, ensured a thorough and rigorous evaluation. Results of the evaluation phase are reported in the Results section.

### Examining the relation between pre‐service teachers' self‐efficacy and their use of motivational messages with human annotations

All analyses were run using Mplus 8.10 (Muthén & Muthén, 1998–2017). Bivariate correlations were analysed with SPSS Version 29. In our data, 11 participants had missing values for TSE beliefs. We estimated the missing values with Full Information Maximum Likelihood estimation (Dong & Peng, 2013). The sample included no missing values for motivational messages. To test how pre‐service TSE dimensions were related to pre‐service teachers use of motivational messages (RQ2), the frequency of motivational messages used by each participant was determined based on human‐rater annotations. To balance the number of motivational messages used in comparison to words spoken, we divided for each participant the number of messages spoken in each category by the total number of words spoken and multiplied the result by 10,000, following Falcon et al. (2023). This procedure ensured that the number of messages was proportional to the number of words spoken. Then, these values were rounded to the nearest integers. The motivational messages variable is a count variable, meaning it can take any value from zero to infinity. Following Muthén et al. (2016), we analysed the relation between pre‐service TSE and their use of motivational messages by applying count regressions and first tested the model fit for: poisson regression, zero‐inflated poisson regression, the negative binomial model and the zero‐inflated negative binomial model. The zero‐inflated negative binomial models had the best fit based on their lowest AIC and BIC values (see Table C1 in Appendix C; Feng, 2021; Hu et al., 2011). The zero‐inflated negative binomial model is a two‐part model. First, it analyses the probability of not using a motivational message, distinguishing structural zeros from sampling zeros (zero‐inflated logit model). Structural zeros arise from subjects who always produce zeros due to an inherent characteristic, such as a pre‐service teacher who is unaware of a certain type of motivational message. A sampling zero occurs when an event that could happen by chance does not occur within the sample, like a pre‐service teacher who usually uses a certain type of motivational message but did not during our observations (Feng, 2021; Hu et al., 2011). Second, a negative binomial count model examines the frequency of supportive and thwarting motivational messages, including sampling zeros and all positive integers (Cho et al., 2019). In total, we tested six zero‐inflated negative binomial models, each model analysing the relations between a pre‐service self‐efficacy dimension and one motivational message type. Against the theoretical background explaining that the three TSE dimensions represent the construct of TSE (Tschannen‐Moran & Hoy, 2001), we correlated the dimensions in the six zero‐inflated negative binomial models. We tested TSE dimensions for multicollinearity, and all had VIF values below 10, indicating no multicollinearity. Figure A2 in Appendix A illustrates the pipeline for addressing the second research question.

### Examining the relation between pre‐service teachers' self‐efficacy and their use of motivational messages with the LLM's predictions

We conducted an additional analysis examining the relation between the LLM's predictions of supportive messages and pre‐service TSE. To achieve this, we used the fine‐tuned model to predict supportive messages for the 103 participants, calculated ratio scores relative to the number of words used, and analysed how these supportive message ratio scores related to pre‐service TSE. We included only the predictions of supportive messages in the analysis, as the LLM did not reliably assess thwarting messages during model evaluation (see Results section). To determine the best‐fitting regression model, we compared poisson regression, zero‐inflated poisson regression, the negative binomial model and the zero‐inflated negative binomial model. The negative binomial model yielded the lowest AIC and BIC values. Subsequently, we analysed three negative binomial regressions for each category of supportive messages in relation to the pre‐service TSE dimensions. The pre‐service TSE dimensions were correlated.

## RESULTS

### Descriptive statistics for pre‐service teachers' human‐rated SDT‐based motivational message use

In total, pre‐service teachers used 2983 instances of motivational messages in class. Autonomy‐supportive messages were used in 31.71% of the labelled messages, 29.47% of the messages were competence‐supportive messages and 21.49% of the messages were relatedness‐supportive messages. In contrast, 9.59% of the messages were autonomy‐thwarting messages, 1.91% were competence‐thwarting messages and 5.83% were relatedness‐thwarting messages. Descriptive statistics and reliability for SDT‐based motivational messages are reported in Table 3.

**TABLE 3:** Descriptive statistics for pre‐service teachers' human‐rated SDT‐based motivational message use.

Motivational message	Total instances of motivational messages across all participants		Participants with at least one instance of a motivational message		Inter‐rater‐reliability	
	*N*_messages_	%	*N*_participants_	%	IRA (%)	Brennan‐ Prediger kappa *κ*
Autonomy‐support	946	31.71	121	88.32	78.79	.72
Competence‐support	879	29.47	110	80.29	72.51	.63
Relatedness‐support	641	21.49	119	86.86	83.15	.78
Autonomy‐thwarting	286	9.59	86	62.77	77.65	.70
Competence‐thwarting	57	1.91	29	21.17	74.07	.65
Relatedness‐thwarting	174	5.83	70	51.09	70.97	.61

*Note*: Instances of motivational messages = Frequency of messages used; IRA (%) = Inter‐rater agreement in percentage (two human‐raters); κ = Brennan–Prediger kappa.

### Evaluation of the Model's performance

Lastly, we evaluated the fine‐tuned model on the given test data set. Our model achieved an average accuracy of 74.80% (±1.20%) across all categories. Table 4 shows the results of the final evaluation phase. While accuracy measures the proportion of correctly classified instances, precision, recall and F1‐scores provide a more detailed evaluation of the model's performance. The message categories of autonomy support, competence support, relatedness support and out‐of‐domain data resulted in F1‐scores over 0.70, indicating robust performance in classifying supportive messages and out‐of‐domain data. However, the message categories of autonomy thwarting, competence thwarting and relatedness thwarting reached F1‐scores below 0.70.

**TABLE 4:** The fine‐tuned model's average performance.

Motivational messages	F1‐score	Precision	Recall	Specificity
Autonomy support	0.73 (±0.01)	0.77 (±0.05)	0.71 (±0.03)	0.94 (±0.02)
Competence support	0.80 (±0.03)	0.81 (±0.07)	0.79 (±0.06)	0.95 (±0.02)
Relatedness support	0.79 (±0.04)	0.79 (±0.06)	0.80 (±0.03)	0.96 (±0.01)
Autonomy thwarting	0.57 (±0.06)	0.55 (±0.08)	0.62 (±0.09)	0.96 (±0.01)
Competence thwarting	0.27 (±0.16)	0.42 (±0.17)	0.22 (±0.14)	1.00 (±0.00)
Relatedness thwarting	0.61 (±0.05)	0.60 (±0.07)	0.63 (±0.05)	0.98 (±0.01)
Out‐of‐domain data	0.78 (±0.01)	0.77 (±0.02)	0.79 (±0.02)	0.88 (±0.02)

*Note*: The model's average performance across five runs.

### Relations between pre‐service teacher self‐efficacy and human‐rated motivational messages

#### Bivariate correlations between pre‐service teacher self‐efficacy and motivational messages

Bivariate correlations are reported in Table 5 and revealed that pre‐service TSE for classroom management and pre‐service TSE for student engagement were positively and significantly associated with the frequency of using relatedness‐supportive messages in class. No other significant associations between pre‐service TSE dimensions and the frequency of other types of motivational messages were found.

**TABLE 5:** Spearman's rank correlations for pre‐service teachers' self‐efficacy and motivational messages.

	TSEIS	TSECM	TSESE	SA	SC	SR	TA	TC	TR
TSEIS	–								
TSECM	.43**	–							
TSESE	.53**	.48**	–						
SA	−.02	.10	.16	–					
SC	−.13	−.07	−.07	.02	–				
SR	.16	.33**	.23*	.23*	−.20	–			
TA	.16	.11	.05	.10	.01	.09	–		
TC	.10	.18	−.10	.00	.08	.03	.27**	–	
TR	.11	.13	−.02	.12	.03	−.02	.39**	.27**	–

Abbreviations: Frequency, Frequency of messages in ratio to words used; SA, Frequency of autonomy‐supportive messages; SC, Frequency of competence‐supportive messages; SR, Frequency of relatedness‐supportive messages; TA, Frequency of autonomy‐thwarting messages; TC, Frequency of competence‐thwarting messages; TR, Frequency of relatedness‐thwarting messages; TSEIS, Teacher self‐efficacy for instructional strategies; TSECM, Teacher self‐efficacy for classroom management; TSESE, Teacher self‐efficacy for student engagement.

* *p* < .05.

** *p* < .01.

#### Longitudinal relations between pre‐service teacher self‐efficacy and motivational messages

In the following, we report only significant effects of the six zero‐inflated negative binomial models. All effect sizes are reported in Table 6.

**TABLE 6:** Results of the zero‐inflated negative binomial models of pre‐service teachers' self‐efficacy and use of motivational messages.

	Negative binomial count models					Zero‐inflated logit models				
	β	*SE*	*p*	95% CI	Exp(β)	β	*SE*	*p*	95% CI	Exp(β)
Autonomy support										
TSEIS	**−0.32**	**0.13**	**0.012**	**[−0.58, −0.07]**	**0.72**	0.13	0.42	0.766	[−0.70, 0.96]	1.13
TSECM	0.07	0.10	0.484	[−0.12, 0.26]	1.07	−0.42	0.53	0.435	[−1.46, 0.63]	0.66
TSESE	0.26	0.17	0.126	[0.07, 0.60]	1.30	−0.25	0.64	0.702	[−1.50, 1.01]	0.78
Competence support										
TSEIS	−0.04	0.22	0.859	[−0.47, 0.39]	0.96	−0.19	0.55	0.723	[−1.27, 0.88]	0.82
TSECM	0.22	0.14	0.121	[−0.06, 0.50]	1.24	0.60	0.43	0.163	[−0.24, 1.44]	1.82
TSESE	−0.36	0.28	0.195	[−0.90, 0.18]	0.70	−0.15	0.52	0.770	[−1.17, 0.86]	0.86
Relatedness support										
TSEIS	−0.17	0.13	0.176	[−0.42, 0.08]	0.84	−0.88	0.56	0.115	[−1.80, 0.22]	0.41
TSECM	**0.38**	**0.13**	**0.003**	**[0.13, 0.63]**	**1.46**	−0.35	0.46	0.449	[−1.09, 0.55]	0.71
TSESE	0.01	0.19	0.952	[−0.35, 0.38]	1.01	0.13	0.58	0.825	[−0.82, 1.26]	1.14
Autonomy thwarting										
TSEIS	0.15	0.25	0.531	[−0.33, 0.64]	1.17	−0.31	0.46	0.508	[−1.21, 0.60]	0.74
TSECM	0.13	0.29	0.664	[−0.44, 0.70]	1.13	−0.11	0.45	0.805	[−0.98, 0.77]	0.89
TSESE	−0.21	0.18	0.263	[−0.57, 0.15]	0.81	0.00	0.51	0.994	[−1.00, 1.00]	1.00
Competence thwarting										
TSEIS	−0.39	0.67	0.565	[−1.70, 0.93]	0.68	−0.95	0.64	0.137	[−2.19, 0.30]	0.39
TSECM	0.19	0.51	0.714	[−0.82, 1.19]	1.21	−0.93	0.57	0.101	[−2.05, 0.18]	0.39
TSESE	0.59	0.41	0.149	[−0.21, 1.39]	1.80	**1.79**	**0.61**	**0.003**	**[0.59, 2.99]**	**5.99**
Relatedness thwarting										
TSEIS	−0.32	0.29	0.266	[−0.88, 0.24]	0.73	−0.95	0.49	0.054	[−1.91, 0.02]	0.39
TSECM	0.09	0.17	0.609	[−0.25, 0.43]	1.09	−0.52	0.41	0.199	[−1.32, 0.27]	0.59
TSESE	0.19	0.27	0.493	[−0.35, 0.72]	1.20	**1.14**	**0.53**	**0.032**	**[0.10, 2.19]**	**3.13**

*Note*: Analyses were carried out with the robust MLR‐estimator. Statistically significant results are shown in bold. TSEIS = Teacher Self‐Efficacy for Instructional Strategies, TSECM = Teacher Self‐Efficacy for Classroom Management, TSESE = Teacher Self‐Efficacy for Student Engagement.

Abbreviations: TSEIS, Teacher Self‐Efficacy for Instructional Strategies; TSECM, Teacher Self‐Efficacy for Classroom Management; TSESE, Teacher Self‐Efficacy for Student Engagement.

##### Pre‐service teachers' self‐efficacy for instructional strategies

Pre‐service TSE for instructional strategies (T_1_) was significantly and negatively related to the frequency of autonomy‐supportive messages (T_2_) used (H2a). High pre‐service TSE for instructional strategies (T_1_) decreased the frequency of using autonomy‐supportive messages (T_2_) in class (Exp(β) = 0.72, *p* = .012).

##### Pre‐service teachers' self‐efficacy for classroom management

Pre‐service TSE for classroom management (T_1_) was significantly and positively related to the frequency of relatedness‐supportive messages (T_2_) used (H2b). High pre‐service TSE for classroom management (T_1_) increased the frequency of using relatedness‐supportive messages (T_2_) in class (Exp(β) = 1.46, *p* = .003).

##### Pre‐service teachers' self‐efficacy for student engagement

Pre‐service TSE for student engagement (T_1_) was significantly positively related to the likelihood of never using a competence‐ or relatedness‐thwarting message (T_2_) (H2c). Pre‐service teachers with higher self‐efficacy for student engagement (T_1_) were more likely to never use a competence‐ (Exp(β) = 5.99, *p* = .003) or relatedness‐thwarting message (T_2_) (Exp(β) = 3.13, *p* = .032).

### Additional analysis on relations between pre‐service teacher self‐efficacy and LLM's predictions of supportive messages

The results of the analysis of the LLM's prediction of supportive messages in relation to pre‐service TSE are presented in Table D1 in Appendix D. In the following, we only report the significant results.

In the LLM's prediction, pre‐service TSE for instructional strategies (T_1_) was significantly and negatively related to the frequency of relatedness‐supportive messages (T_2_) used. High pre‐service TSE for instructional strategies (T_1_) decreased the frequency of using relatedness‐supportive messages (T_2_) in class (Exp(β) = 0.71, *p* = .001). Furthermore, pre‐service TSE for student engagement (T_1_) was significantly and positively related to the frequency of relatedness‐supportive messages (T_2_) used. High pre‐service TSE for student engagement (T_1_) increased the frequency of using relatedness‐supportive messages (T_2_) in class (Exp(β) = 1.32, *p* = .026).

## DISCUSSION

In the study, we aimed to fine‐tune an LLM to reliably assess pre‐service teachers' SDT‐based motivational messages. We also aimed to test the relations between different dimensions of pre‐service TSE and the use of motivational messages. The fine‐tuned LLM performed well in classifying pre‐service teachers' motivational messages in four of the seven categories. Additionally, our findings with the human‐rated annotations showed that pre‐service TSE for classroom management positively affected the frequency of relatedness‐supportive messages, while pre‐service TSE for instructional strategies negatively affected the frequency of autonomy‐supportive messages. Moreover, pre‐service TSE for student engagement increased the likelihood of never using a competence‐ or relatedness‐thwarting message. The following sections discuss the results in detail.

### Fine‐tuning and evaluation of an LLM to assess teachers' motivational messages

In our study, the categories of autonomy support, competence support, relatedness support and out‐of‐domain data obtained an F1‐score above 0.70, indicating robust classification performance compared to studies in a similar domain (Falcon & León, 2024; Tan & Demszky, 2023). However, the fine‐tuned LLM did not reach high F1‐scores for classifying thwarting messages. We identified two challenges that may have contributed to reduced effectiveness in classifying thwarting messages. The first challenge is the imbalance of thwarting messages in the training set. Pre‐service teachers used thwarting messages five times less frequently than supportive messages, resulting in the LLM not being trained with an equal amount of data across all categories. Augmenting the training data with AI‐generated messages did not mitigate this issue. For further research, we suggest using a large and balanced data set of authentic classroom messages to achieve high accuracy in all categories. Additionally, if the data set is augmented with synthetic data through AI‐generated messages, we recommend incorporating a few examples of the original messages in the instructional prompt (few‐shot approach) to ensure that the synthetic examples more closely align with the linguistic patterns of authentic classroom data.

The second challenge we identified concerns the quality of the training data in terms of its distinctiveness. LLMs are proficient in recognising data patterns; however, if the data exhibit high variability, the LLM may struggle to identify all linguistic patterns (Munappy et al., 2022). For instance, during the initial human annotation process, the raters used 23 sub‐categories, which were later consolidated into six main SDT‐based motivational message categories. The message data from these six main categories were used to fine‐tune the LLM. However, due to the initial annotation involving 23 sub‐categories, the LLM encountered a wide range of sub‐themes and sub‐patterns within the six main categories, which may have contributed to lower model performance. For future research, we recommend using clearly distinctive categories with consistent examples that exhibit low variability. A more detailed discussion of these challenges can be found in Metzner et al. (2025).

### Relations between pre‐service teacher self‐efficacy and human‐rated motivational message use

Our expectations about the relations between pre‐service TSE and their use of SDT‐based motivational messages were partially met. We did not find the assumed significant relations between pre‐service TSE for instructional strategies and pre‐service teachers' use of competence‐supportive messages in class mid‐semester (T_2_) (H2a). One possible explanation for this could be the complexity involved in delivering competence‐supportive messages. Specifically, in the context of providing constructive feedback – a component of competence‐supportive messages in our classification system – research has demonstrated that teaching experience is positively related to the frequency and quality of feedback (Holstein et al., 2022). Our sample included only pre‐service teachers, and thus the lack of years of teaching experience might be an explanation for the insignificant relation between their self‐efficacy beliefs and their use of competence‐supportive messages.

However, our results showed that pre‐service teachers with high self‐efficacy for instructional strategies used autonomy‐supportive messages significantly less frequently, which is in line with our expectations (H2a). One explanation for this finding could be that pre‐service teachers with high self‐efficacy for instructional strategies tend to emphasise strategies that promote learners' thinking through performance diagnostics and adjustments, such as implementing variousmanagement approach that conc assessment strategies or providing appropriate challenges for learners (Tschannen‐Moran & Hoy, 2001). Conversely, this could mean that pre‐service teachers with high self‐efficacy for instructional strategies prioritise creating an adaptive learning environment over implementing participative practices, such as autonomy‐supportive messages in class. Moreover, pre‐service TSE for instructional strategies was not significantly related to the use of relatedness‐supportive messages, which is in line with our expectations (H2a).

Supporting our assumptions (H2b), our results showed a significant positive relation between pre‐service TSE for classroom management and the frequency of relatedness‐supportive messages. This finding aligns with Pianta's (2006) interpersonal classroom management approach that conceptualises classroom management as a dynamic process that describes positive teacher–student interactions as an inherent part of teachers' classroom management strategies enabling positive student behaviour and preventing misbehaviour (Pianta et al., 2012). Building on this approach, positive relations between pre‐service TSE for classroom management and these pre‐service teachers' use of relatedness‐supportive messages can be understood as a dynamic teacher management process, where classroom interactions promote a relatedness‐supportive learning environment.

Consistent with our assumptions (H2c), our findings revealed that pre‐service teachers with high self‐efficacy for student engagement were significantly more likely to never use a relatedness‐thwarting message. Additionally, they were also significantly more likely to never use a competence‐thwarting message, which we did not expect. Putwain and von der Embse (2018) hypothesised that in classes with unmotivated students, teachers with lower self‐efficacy beliefs for student engagement resort to more controlling teaching strategies, such as the use of fear appeals to engage students. In our study, we did not include student motivation data, but found a similar relation between high pre‐service TSE for student engagement and the likelihood of not using a competence‐ or relatedness‐thwarting message. This implies that pre‐service teachers' beliefs about their ability to motivate students affect the probability that they will not use controlling language in class and they in particular are more likely to never use a competence‐ or relatedness thwarting message. However, pre‐service TSE for student engagement was not related to their use of autonomy‐ or relatedness‐supportive messages, which did not confirm our expectations (H2c). One possible explanation for this could be that the use of autonomy‐ and relatedness‐supportive messages could depend on, besides TSE, the quality of the teacher–student relationship. Research has shown that students' perceptions of the teacher–student relationship are longitudinally and positively related to their perceived autonomy and sense of relatedness in class (Bakadorova & Raufelder, 2018). In our study, pre‐service teachers met the students only once and thus had limited time to learn students' individual interests, needs and motivation – factors essential for building effective teacher–student relationships (Pianta et al., 2012).

On a theoretical level, our findings imply that – in line with Tschannen‐Moran et al.'s (1998) model – TSE is indeed affecting teachers' effort and behaviour in teaching situations. Our contribution to theory development is that we assessed teachers' verbal messages not through their own or students' self‐reports, but through objectively assessing their verbal behaviours using classroom video data. Thus, motivational messages were shown in this study to be predicted by teachers' TSE dimensions, and thus could be a link from teachers' TSE to student motivation. However, as we did not have data on student motivation, we were only able to examine one side of this link. On a theoretical level, the underlying mechanism that may explain the significant effect of TSE dimensions on SDT‐based motivational messages in our study could be that teachers who believe that they are able to motivate students also invest effort in doing so, and this is displayed in their language use in class. However, theoretically, to further validate this mechanism, analyses are needed in future research that include data on teachers' behavioural effort (e.g., time spent with unmotivated students in class using video data) as well as student data on student motivation as an outcome.

### Relations between pre‐service teacher self‐efficacy and the LLM's predictions of supportive messages

We conducted an additional analysis examining the relation between pre‐service TSE and the LLM's predictions of supportive messages. The results revealed different relations between pre‐service TSE and supportive message use compared to the regressions based on human coding. The analysis did not confirm the previously found path between pre‐service TSE for instructional strategies and a lower use of autonomy‐supportive messages. In the LLM‐based analyses, pre‐service TSE for instructional strategies impeded the likelihood of using relatedness‐supportive messages. Thus, apparently, considering both sets of analyses, self‐efficacy for instructional strategies cannot be considered as a relevant motivational factor contributing to need‐supportive teachers' messages use.

Analyses based on human annotations showed that pre‐service TSE for student engagement increased the likelihood of never using a competence‐ or relatedness‐thwarting message. In the LLM‐based analyses, we were not able to include the thwarting messages because of their low reliability, but extending the findings based on human annotations, LLM‐based analyses showed that pre‐service TSE for student engagement was positively related to the use of relatedness‐supportive messages, which aligns with our expectations (H2c). We assume that these results may be due to the LLM incorporating additional relatedness‐supportive patterns in the coding that were not included in the original coding scheme. For example, a closer examination of the LLM's predictions of supportive messages revealed that the model classified phrases such as “Thank you for your writings. I really liked them.” as relatedness‐supportive messages. However, the original coding scheme did not include student praise unless it explicitly referred to a specific course of action. A further analysis of the LLM's predictions regarding autonomy‐supportive messages reveals that the LLM annotated sentences such as “Did the author capture the image well, or do you think this image is more suitable?” as an autonomy‐supportive message. This sentence could reflect pre‐service teachers incorporating students' perspectives; however, in the initial coding scheme, we did not classify these kinds of messages as autonomy‐supportive. In conclusion, the regression analysis using data from both human annotations and LLM predictions has shown that relatedness‐supportive messages are associated with pre‐service TSE, but the results vary slightly depending on the rater (human vs. LLM).

Taken together, although patterns of analyses based on human codings and based on LLM differed, the findings suggest that the LLM predictions may reveal additional meaningful insights. For instance, the relation between pre‐service TSE for instructional strategies and relatedness‐supportive messages in the human‐coded data also showed a negative association, which was not statistically significant (β = −0.17, *p* = .176). This indicates that the association was present within both the LLM and human‐coded data but could not be fully uncovered based solely on the annotated material. Additionally, as discussed earlier, the original coding scheme was complex due to a variety of sub‐themes and sub‐patterns. During the initial coding process, human raters could rely on contextual information from the broader classroom discourse to categorise messages. This ability may lead to more nuanced decisions, whereas the LLM might make more immediate classifications. Consequently, this could result in the LLM classifying more sentences as supportive messages overall due to its lack of access to broader contextual information.

### Limitations and future directions

Regarding our first research question, the message categories of supportive messages and out‐of‐domain data obtained F1‐scores above 0.70, indicating that the fine‐tuned LLM can effectively classify these messages in a resource‐efficient manner. However, the low frequency of thwarting messages among pre‐service teachers led to lower model performance in detecting these specific message types. We recommend using a balanced training set across all message categories to enhance the performance of LLMs in classifying SDT‐based messages from teachers, especially for those from minority classes, such as thwarting messages. Additionally, if training data augmentation with synthetic AI‐generated messages is considered to expand the data set, we recommend using a few‐shot approach. Moreover, we want to acknowledge that, in our study, the model's training data are situated in a specific context (i.e., pre‐service teachers with limited teaching experience). To ensure that LLMs are robust, one should test their performance in varied contexts, such as in samples of both pre‐service and in‐service teachers or with regard to specific school subjects, to verify that model predictions remain accurate across diverse teaching environments Wang et al., 2024. Given the large number of resources needed to classify teachers' messages using human raters in research processes, the LLM‐based approach offers a promising, though not yet well‐developed, pathway to more efficient and reliable assessments of teacher–student speech and dialogues in classroom settings. This approach can provide deeper insights into social dynamics and motivational processes that have remained largely unexplored. Going beyond student self‐reports to assess such messages, the LLM method provides a high degree of objectivity, allowing researchers to validly assess classroom interactions while minimising the risk of common‐method bias (Podsakoff et al., 2024) that may occur when both motivational messages and student outcomes rely solely on student ratings. Therefore, we believe LLMs hold significant potential to shape future educational research; however, further investigation is needed to fully understand their strengths and challenges. With regard to teaching practice, the use of a fine‐tuned LLM has the potential to serve as an automated feedback tool for teachers regarding their use of instructional or motivational messages. This could enable instructors to independently monitor their speech and repeatedly receive feedback on the messages they employ (Demszky et al., 2024; Reeve & Cheon, 2021).

Regarding our second research question, the sample size of pre‐service teachers considered was comparatively small, which led to low statistical power for testing. However, despite the suboptimal statistical power, we were still able to identify substantial effects of pre‐service TSE on their motivational messages. Further studies are needed to validate our findings using larger data sets. Additionally, our study did not account for the speaker's tone of voice, although research has shown that a controlling tone of voice increases listeners' sense of pressure, decreases their willingness to cooperate and creates a sense of distance, regardless of the message (Vrijders et al., 2024). In this study, we focused on pre‐service teachers in a Bachelor programme. Although it could be assumed that pre‐service teachers have limited practical experiences and thus their self‐efficacy beliefs might not realistically reflect their skills (for further discussion, see Klassen & Chiu, 2011), prior work has shown that pre‐service TSE for student engagement positively predicts observer‐rated emotional support during practical teaching experiences in schools (Hußner et al., 2024). Thus, it can be assumed that even in early stages of pre‐service teachers' studies, their self‐efficacy already affects how they teach in class. Moreover, the pre‐service teachers in our study participated in a course on motivation‐enhancing teaching practices before teaching a lesson in a secondary school. The specific study context and the course materials they engaged with prior to teaching may have influenced their instructional practices and the motivational messages they employed in the class. To gain a clearer understanding of how pre‐service TSE affects the motivational messages these pre‐service teachers use, a control group of pre‐service teachers who have not engaged with course materials on motivation‐enhancing teaching would be required.

## CONCLUSION

The fine‐tuned LLM demonstrated promising performance in classifying supportive messages and out‐of‐domain data, suggesting it is a reliable tool for assessing pre‐service teachers' SDT‐based motivational messages. However, for the thwarting messages, the model's performance was insufficiently robust. Additionally, using the human annotations and the LLM predictions of motivational messages, our study demonstrated relations between pre‐service TSE beliefs and their use of motivational messages. We showed that high pre‐service TSE does not automatically relate to the likelihood and frequency of using motivational messages; rather, it depends on the specific dimension of self‐efficacy, which will affect the dimensions of supportive and thwarting messages differently. Our research has practical relevance as the LLM‐based models of teacher motivational messages can be leveraged in teacher education, school practice and teacher training to develop personalised feedback tools for teachers that are evidence‐based and tailored to individual in‐class behaviours.

## AUTHOR CONTRIBUTIONS

**Olivia Metzner:** Conceptualization; methodology; data curation; investigation; formal analysis; project administration; resources; writing – original draft; writing – review and editing. **Yindong Wang:** Formal analysis; investigation; methodology; software; writing – original draft; writing – review and editing. **Wendy Symes:** Writing – review and editing. **Yizhen Huang:** Writing – review and editing. **Lena Keller:** Formal analysis; writing – review and editing. **Gerard de Melo:** Writing – review and editing. **Rebecca Lazarides:** Conceptualization; funding acquisition; investigation; methodology; project administration; resources; supervision; writing – original draft; writing – review and editing.

## CONFLICT OF INTEREST STATEMENT

The authors declare no conflicts of interest.

## Supporting information


Data S1:

## Data Availability

The data that support the findings of this study are available on request from the corresponding author. The data are not publicly available due to privacy or ethical restrictions.

## APPENDIX B

### B.1 Initial classification experiments with LLMs

Prior to the fine‐tuning experiments with the LLM Gemma 2 27B (Gemma Team, 2024), we conducted several experiments with various open‐source LLMs. First, we explored in‐context learning using the model LeoLM/leo‐mistral‐hessianai‐7b‐chat (Plüster, 2023) and provided the model selected examples in a prompt to classify six labels. This approach yielded an accuracy of 0.27 and an F1‐score of 0.18 in the 3‐shot setting. Additionally, we experimented with a two‐stage few‐shot classification approach, where the model first distinguished between supportive and thwarting messages and then classified them as related to autonomy, competence or relatedness. For this approach, we used the DeBERTA‐v3‐large model (He et al., 2021) and obtained an accuracy of 0.53 and an F1‐score of 0.49 in the first stage. In the second stage, we achieved an accuracy of 0.68 and an F1‐score of 0.68. Furthermore, we tested fine‐tuning methods with various LLM sizes, including Gemma‐7B (Banks & Warkentin, 2024), achieving an accuracy of 72.27% and an F1‐score of 0.64.

## APPENDIX C

### C.1 Comparison of the model fits

**TABLE C1:** Summary of count regression model fits for RQ2.

	LL	Par	AIC	BIC	LL	Par	AIC	BIC	LL	Par	AIC	BIC
	Autonomy‐supportive messages				Competence‐supportive messages				Relatedness‐supportive messages			
1	−1202.00	13	2430.00	2464.25	−1574.27	13	3174.53	3208.79	−1024.31	13	2074.62	2108.87
2	−984.09	17	2002.17	2046.96	−1219.24	17	2472.47	2517.26	−876.94	17	1787.88	1832.67
3	−669.56	14	1367.13	1404.01	−637.03	14	1302.06	1338.95	−627.27	14	1282.53	1319.42
4	−646.09	18	1328.18	1375.61	−624.70	18	1285.40	1332.82	−612.89	18	1261.79	1309.21
	Autonomy‐thwarting messages				Competence‐thwarting messages				Relatedness‐thwarting messages			
1	−861.74	13	1749.47	1783.72	−498.73	13	1023.46	1057.71	−848.57	13	1723.13	1757.39
2	−621.86	17	1277.72	1322.51	−335.43	17	704.85	749.64	−562.91	17	1159.81	1204.60
3	−526.36	14	1080.73	1117.61	−330.74	14	689.48	726.36	−481.90	14	991.79	1028.68
4	−514.11	18	1064.23	1111.65	−321.47	18	678.93	726.36	−467.44	18	970.88	1018.31

*Note*: 1 = Poisson regression, 2 = Zero–inflated poisson regression, 3 = Negative binomial model, 4 = Zero‐inflated negative binomial model.

Abbreviations: AIC, Akaike information criterion; BIC, Bayesian information criterion; LL, Log‐likelihood; Par, Quantity of parameters.

## APPENDIX D

**TABLE D1:** Results of the negative binomial models of pre‐service teachers' self‐efficacy and use of LLM predicted supportive messages.

	β	*SE*	*p*	95% CI	Exp(β)
Autonomy support					
TSEIS	0.06	0.08	0.413	[−0.09, 0.21]	1.06
TSECM	0.06	0.06	0.284	[−0.05, 0.18]	1.07
TSESE	−0.10	0.09	0.258	[−0.28, 0.08]	0.90
Competence support					
TSEIS	0.03	0.10	0.748	[−0.16, 0.22]	1.03
TSECM	−0.02	0.10	0.870	[−0.21, 0.17]	0.98
TSESE	−0.02	0.12	0.867	[−0.25, 0.21]	0.98
Relatedness support					
TSEIS	**−0.35**	**0.11**	**0.001**	**[−0.55, −0.14]**	**0.71**
TSECM	0.05	0.08	0.497	[−0.10, 0.20]	1.05
TSESE	**0.28**	**0.12**	**0.026**	**[0.03, 0.52]**	**1.32**

*Note*: Analyses were carried out with the robust MLR‐estimator. Statistically significant results are shown in bold.

Abbreviations: TSEIS, Teacher Self‐Efficacy for Instructional Strategies; TSECM, Teacher Self‐Efficacy for Classroom Management; TSESE, Teacher Self‐Efficacy for Student Engagement.
