# Supplementary material for: A process‐oriented perspective on pre‐service teachers' self‐efficacy and their motivational messages: Using large language models to classify teachers' speech
Source: Br J Educ Psychol. 2025 Apr 28;95(Suppl 1):S73–97. doi: 10.1111/bjep.12779 (PMC12427170; doi:10.1111/bjep.12779)
Supplement: Supplementary file 1 — Data S1: [file BJEP-95-S73-s001.docx]

# Supplemental material for the paper “A Process-Oriented Perspective on Pre-Service Teachers’ Self-Efficacy and Their Motivational Messages: Using Large Language Models to Classify Teachers’ Speech”

## Supplement A: Coding Scheme—SDT-Based Motivational Messages

**Table 1**

*Supportive Motivational Messages*

| Main categories | Definition | Sub-categories | Examples |
| --- | --- | --- | --- |
| Autonomy-supportive messages | Teacher includes possibilities for participation within the lesson. The learners are able to influence or choose the content, tasks, methods, or pace of learning. The teacher explains the reasoning behind the tasks, methods, content, or learning goals, and makes clear what the performance expectations are. The learners give the teacher feedback about their learning experiences. The teacher makes additional learning materials available, which the learners are able to work on voluntarily. | Participation and opportunities to make choices regarding: contents, methods  social forms, time | “I have a task-pool of four tasks of which you can choose one to work on.”  “You can choose how much time you want to take for each task.” |
|  |  | Explaining the reasoning behind tasks/methods/ content/learning goals/performance expectations | “One reason for us covering this in class is that you’ll frequently need to calculate percentages in everyday life – for example, to calculate how many Euros will be discounted, if a price is reduced by 10%.”  “In the following task I expect you to write full sentences, not bullet-points.” |
|  |  | Asking learners about their learning experience | “Please open the Flinga board and write down some key words about what you liked about this teaching unit and what you found to be less interesting.” |
|  |  | Additional learning materials to work on voluntarily | “Here are some additional tasks if you want to practice at home voluntarily.” |
| Competence-supportive messages | The teacher creates a learning environment in which students feel capable and competent. The teacher uses encouraging language and gives constructive feedback. The teacher asks about the learners’ opinions and views and asks questions which enable a deeper engagement with the topic. | Giving prompt, concrete, positive feedback  relating to:  - the correctness of the answer/solution to the task  - the content and/or strategic assistance for the working process | “You could also use a different formula for this task, which would get you to the solution faster.”  “This answer was very good, because it shows how to reach the solution in clear steps.” |
|  |  | Hope, encouragement, and optimism relating to the competences and abilities of the learner | “I know this is a difficult task, but you can do it.” |
|  |  | Questions that enable a deeper understanding of the content and that follow up on the opinions/views of the learners | “Can you evaluate the differences between the two epochs?”  “Do you have another idea why the character in the novel acted like that?” |
| Relatedness-supportive messages | The teacher creates an appreciative and accepting learning environment. The teacher shows a clear interest in the well-being of the learners and gives appreciative feedback on the social interaction of the class. The teacher encourages the cooperation of the class by exemplifying goals and uses for cooperative work. The teacher presents the content in an enthusiastic manner. The teacher displays understanding for learners’ views and encourages these. | Questions about well-being | “How are you feeling today?” |
|  |  | Appreciative feedback about the social climate | “You are a very kind class; I think it’s great how you are working through the topic together with me.”  “Thanks for your participation.” |
|  |  | Promoting cooperation | “You can solve this task as a group and support each other with the work.”  “Work together with your neighbor and communicatively find out the main aspects of the text.” |
|  |  | Teacher enthusiasm | “The next part of this teaching unit is really interesting.” |
|  |  | Showing understanding for the learners’ viewpoints | “I understand that there are more fun things you would rather do in your free time.” |
|  |  | Assistance and encouragement | “It's not terrible if it isn’t perfect.”  “Don’t worry.”  “If you have any questions, you can come to me any time.” |

**Table 2**

*Thwarting Motivational Messages*

| Main category | Definition | Sub-categories | Examples |
| --- | --- | --- | --- |
| Autonomy-thwarting messages | The teacher creates a learning environment in which learners feel put under pressure and controlled in their actions. The teacher uses pressuring language in class and strict deadlines. The teacher doesn’t let the learners find solutions to questions themselves and includes tasks in their lessons which exclude individual learners. | Use of pressuring language | “You should be able to explain this better.” |
|  |  | Tasks which exclude individual learners | “Only those who did their homework may play a game now.” |
|  |  | Setting pressuring deadlines | “You only have another two minutes for this task.”  “The lesson will be over soon, so please quickly finish this task” |
|  |  | Pre-providing solutions or answers | Teacher: “Who has the answer to task 4?” No learner answer. Teacher: “The answer is 42.” |
| Competence-thwarting messages | Teacher creates a learning environment in which learners feel unable to complete tasks. The teacher criticizes fixed attributes of the learners and does not give constructive criticism. The performances of the learners are being compared to those of their classmates. | Criticizing of a fixed attribute | “Maths is not your strong suit.” |
|  |  | Unfriendly, destructive criticism | “You should be able to do that better” |
|  |  | Competitive comparisons with classmates, e.g., through performance comparisons | “Julia, you have the worst results out of everyone.”  “You should learn from Paula, who got the most points on the test.” |
| Relatedness-thwarting messages | Teacher creates a learning environment in which learners feel rejected, judged, and degraded. The teacher uses insulting and derogatory language and a harsh tone. The teacher is sarcastic/ironic towards the learners. | Using insulting or derogatory language/contents/curse words | “Your idiotic behaviour got you there!” |
|  |  | Shouting or using a harsh tone | “Hey, just be quiet now!” |
|  |  | Sarcasm/irony | “Great, you finally understood it” |
